# Consumption of Meat and Dairy Products Is Not Associated with the Risk for Rheumatoid Arthritis among Women: A Population-Based Cohort Study

**DOI:** 10.3390/nu11112825

**Published:** 2019-11-19

**Authors:** Björn Sundström, Lotta Ljung, Daniela Di Giuseppe

**Affiliations:** 1 Department of Public Health and Clinical Medicine, Rheumatology, Umeå University, 90187 Umeå, Sweden; lotta.ljung@umu.se; 2 Clinical Epidemiology Division, Department of Medicine Solna, Karolinska Institutet, 171 77 Solna, Sweden; daniela.digiuseppe@ki.se

**Keywords:** diet, animal origin, meat, dairy products, rheumatoid arthritis

## Abstract

Diet has gained attention as a risk factor for the development of rheumatoid arthritis (RA), especially with regards to food of animal origin, such as meat and dairy products. By using data from national patient registers and dietary data from a large prospective population cohort, the Swedish Mammography Cohort, we aimed to investigate whether the consumption of meat and dairy products had any impact on the risk of subsequent development of RA. During 12 years of follow-up (January 2003–December 2014; 381, 456 person-years), 368 patients with a new diagnosis of RA were identified. No associations between the development of RA and the consumption of meat and meat products (hazard ratio [HR] for the fully adjusted model: 1.08 [95% CI: 0.77–1.53]) or the total consumption of milk and dairy products (HR for the fully adjusted model: 1.09 [95% CI: 0.76–1.55]) were observed. In conclusion, in this large prospective cohort of women, no associations were observed between dietary intake of meat and dairy products and the risk of RA development.

## 1. Introduction

Rheumatoid arthritis (RA) is a systemic chronic form of autoimmune arthritis in which both genetic and environmental factors contribute [1,2,3]. It affects between approximately 0.5% and 1% of the population in industrialized countries, with a yearly incidence of between 5 and 50 cases per 100,000 individuals [3]. The most established environmental risk factor for RA is smoking, approximately doubling its risk [4]. Diet, as an inevitable environmental risk factor, has historically received considerable attention and yielded several hypotheses, not the least in advocating vegetarian and vegan diets, thereby indicating that food of animal origin, such as meat and dairy products, may be associated with disease activity, as well as the development of the disease [5].

However, analyses on the consumption of meat and dairy products as risk factors have yielded inconclusive results. The consumption of meat as a risk factor for RA development was scientifically first investigated in an ecological study [6]. Later, a prospective case-control study [7] investigated the risk for inflammatory polyarthritis, not specifically RA. Follow-up studies have failed to verify the initial reports of a detrimental effect of meat in increasing the risk to develop RA or inflammatory polyarthritis [8,9]. Milk and dairy products have been suggested as risk factors, mainly due to the hypersensitive allergic responses in two case reports [10,11] and in a rat model of inflammatory arthritis [12]. However, prospective studies in humans have not been able to verify this; instead, a trend towards an inverse association between the consumption of dairy products and risk for RA has been described [13].

A challenge in evaluating the impact of dietary habits is the availability and access to data before the onset of the disease, since the patients may change their diet after the diagnosis of a chronic disease, and the presence of a chronic disease can induce a bias in the results in a dietary investigation. Only a few large population-based prospective cohorts with data on dietary habits worldwide exist; one of them is the Swedish Mammography Cohort (SMC). Therefore, we aimed to investigate the impact of the consumption of meat and dairy products on the risk for subsequent development of RA in the previously collected data from the SMC.

## 2. Materials and Methods

### 2.1. Study Population and Settings

The prospective SMC started in 1987 when women born between 1914 and 1948 living in the Uppsala and Västmanland counties received a self-administered questionnaire. Altogether, 66,651 women returned the form (response rate: 74%), which contained questions regarding lifestyle, such as alcohol consumption and diet, as well as background data, such as height, weight, parity, and educational level. A second investigation was conducted in 1997 with a questionnaire to update previous data. Also, additional data was collected regarding the use of dietary supplements, smoking history, and physical activity; the questionnaires were sent to all previous participants who were still alive (39,227 participants; response rate 70%).

From the cohort, individuals with incorrect or missing personal identification numbers were excluded (*n* = 243, 0.6%), as well as those who reported unrealistic dietary data in the 1997 investigation (defined as those with a daily energy intake ±3 standard deviations from the mean of the population, *n* = 511, 1.3%). Those diagnosed with RA (International Classification of Diseases, 10th revision [ICD-10] codes M05 and M06) (*n* = 541, 1.5%), other forms of arthritis (ICD-10 codes M07-M12, M14, M45, M46, M30-M36) (*n* = 732, 1.9%), or those who died (*n* = 1600, identified through the Swedish Death Register) after filling the second questionnaire and before the start of follow-up on 1 January 2003 were excluded. The final study cohort included 35,600 women aged 54–89 years at the start of follow-up.

Completion and return of the self-administered questionnaire in SMC constituted the participants’ informed consent. The study and its protocol were reviewed and approved by the Central Ethical Review Board in Stockholm, Sweden (decision no. 2016/2034-31/1) and conducted fully in line with the Declaration of Helsinki.

### 2.2. Identification of the Outcome and Follow-Up of the Cohort

Individuals who developed RA were identified through a new diagnosis of RA in one or two different national registers in Sweden: (1) the National Patient Register administered by the National Board of Health and Welfare, with information on diagnoses coded using the International Classification of Diseases (ICD) from all hospitalizations on national basis since 1987, outpatient visits in non-primary care since 2001, and/or (2) a diagnosis of RA in the Swedish Rheumatology Quality register, which is a clinical register established in the mid-1990s that monitors patients with newly diagnosed early RA, fulfilling classification criteria, as part of standard care. A previous validation using clinical records indicated that approximately 90% of the patients with an RA diagnosis in the National Patient Register fulfilled the American Rheumatism Association 1987 revised classification criteria [14,15]. For linkages between all registers and the SMC, the unique Swedish personal identification number was used [16].

The follow-up started on 1 January 2003 and ended on 31 December 2014. The delay in the start of the follow-up was related to the presence of mixed prevalent and incident cases defined as new cases during the first years of the outpatient register in 2001, as reported previously [17].

### 2.3. Dietary Assessment

Diet was assessed using data from a food-frequency questionnaire (FFQ) containing 67 questions in 1987 (FFQ 1987) and 96 questions in 1997 (FFQ 1997). The FFQ was validated previously [18,19,20]. In the FFQs, participants were asked to specify their average consumption of various foods over the past year on an eight-step predefined scale ranging from never to three times or more per day. The consumption of meat was categorized into processed and unprocessed red meat, poultry, and total meat consumption. Unprocessed red meat included pork, beef/veal, and minced meat; processed included sausages and cold cuts, such as ham/salami, blood pudding, and liver pate; poultry included the stated consumption of chicken/other poultry. The consumption of dairy products was analyzed as consumption of milk, cheese, and total consumption of all above-mentioned dairy products, with the addition of cream and crème fraiche. All variables were categorized in quartiles. Missing values for individual foods were interpreted as no consumption.

### 2.4. Statistical Analyses

All women were followed from baseline (1 January 2003) until the date of first diagnosis of RA, death, or until the end of the follow-up period (31 December 2014), whichever came first. A Cox proportional hazards regression model, with age (in years) as the underlying time scale, was used to estimate hazard ratios (HRs) and their 95% confidence intervals (CI) across categories of dietary intake. Simplified, the HR can be explained as the multiplicator of the rate per time unit for development of the outcome among a population with a certain exposure, compared with the rate per time unit in the comparator population. A HR of 2 thus indicates that twice as many exposed individuals develop the outcome per time unit, compared with the unexposed individuals. Accordingly, an HR of 1 indicates a similar rate of the outcome among exposed and unexposed individuals. The impact of long-term consumption of meat and dairy products was evaluated by using individuals with a consumption below the median both in 1987 and 1997 as a reference. In the multivariable models, adjustments were performed for age (time scale of the model), cigarette smoking status (categorized as never, former, current ≤10 cigarettes per day, or >10 cigarettes per day), alcohol consumption (never, former, current <2 drinks per week, or ≥2 drinks per week), energy intake (categorized as quartiles), fish consumption (quartiles), and dairy or meat consumption. Additional adjustment for educational level was considered, but as shown in previous studies [17,21], education is not a confounder in this population. Statistical analyses were performed using SAS (version 9.4; SAS Institute, Cary, NC, USA) and STATA (version 14, StataCorp, Lakeway, TX, USA). Two-tailed *p*-values ≤ 0.05 were considered significant.

## 3. Results

During the 12-year follow-up period (January 2003–December 2014; 381,456 person-years), 368 new cases with RA were diagnosed. The consumption of meat and dairy products are presented in Table 1 together with the characteristics of the studied population. Consumption of dairy and meat products in the studied cohort was common with approximately 52% consuming meat products more than once per day, and approximately 80% of the population consuming dairy products more than thrice per week.

Consumption of meat and meat products was not associated with the development of RA in age-adjusted (hazard ratio (HR) = 0.96 (95% CI: 0.69–1.32)), or multivariable adjusted models (HR = 1.08 (95% CI: 0.77–1.53; Table 2)). Associations with RA development were neither observed for consumption of type-specific meat, such as red meat (HR = 1.08 (95% CI: 0.77–1.50)), processed meat (HR = 0.84 (95% CI: 0.59–1.22)), or poultry (HR = 0.88 (95% CI: 0.60–1.31)).

No associations were observed between the total consumption of milk and dairy products and the development of RA. The HR for the fully adjusted model was 1.09 (95% CI: 0.76–1.55; Table 3). Moreover, when evaluating milk and cheese consumption as individual groups, no associations with the risk of RA could be observed. The fully adjusted HR for the highest milk consumption was 1.07 (95% CI: 0.80–1.43), and the highest cheese consumption HR was 1.20 (95% CI: 0.81–1.76).

In the long-term analysis, women with a consistent consumption above the median, both during the investigation in 1987 and 1997, of meat and dairy products, did not exhibit an increased risk of developing RA (Table 4).

## 4. Discussion

In this prospective cohort study of women, we could not observe any association between the consumption of meat and dairy products and the risk of RA. Regarding meat, this is in line with results from other prospective cohort studies, such as the Nurse’s Health Study [9] and the Danish Diet, Cancer, and Health cohort [8], which both failed to confirm the finding of an association between meat consumption and the risk for inflammatory polyarthritis observed in the EPIC-NOAR study [7]. Regarding milk, our findings are inconsistent with those of the EPIC-NOAR study [7], which reported a positive, although not statistically significant, association, and the Iowa Women’s Health Study, which reported a weak inverse association [13].

Any unfavorable effect of meat on the development of RA could have several reasons. Perhaps, the most well-known and discussed reason is its content of arachidonic acid, giving rise to the arachidonic acid cascade, which, in turn, gives rise to eicosanoids of mainly pro-inflammatory character, such as PGE-2 [22]. However, the human body has been suggested to endogenously produce a substantial portion of its arachidonic acid content from linoleic acid [23], which implicates that dietary sources and intake of arachidonic acid may be of lesser importance. This could explain the lack of an association in our results. Nitrate and iron are other possible hazardous agents in meat, as both have been suggested to increase the oxidative stress in the body [6,24]. However, regarding iron intake, Pattison et al. described that the association between meat consumption and the risk for arthritis was independent of iron consumption [7]. Benito-Garcia et al. could not find any association between intake of iron and the risk of developing RA [9]. The intake of nitrite is more complicated to evaluate, since nitrite compounds are usually added to processed and cured meat as preservative agents and as color fixatives [25]. This results in a considerable variation in the nitrite content between different products from different manufacturers, despite the legalization of permitted levels in food products [26].

A detrimental effect of milk on both the occurrence of RA and disease activity has been suggested to be associated with an allergic reaction to cow milk protein [11,12,27]. However, in an experiment testing mucosal sensitivity to cow milk protein in patients with RA, mucosal reactivity was observed only in a minor fraction of the patients [28]. A protective effect of milk and dairy products have been suggested to be associated with its nutritious contents of mainly vitamin D [13]. In animal/murine models, high intake/levels of vitamin D has been indicated to suppress the development of arthritis [29]. However, the fact that the biological availability of vitamin D is determined by several factors, of which exposure to sunlight might be of more importance than dietary sources, should be noted. In the analyses of plasma/serum availability of vitamin D before the onset of symptoms in RA, no associations were observed [30,31].

Taken together, little or no evidence exists to suggest that the digestion of food of animal origin is relevant to the pathogenesis of RA. However, it is not uncommon among patients to believe that food of animal origin, such as meat and dairy products, contributed to the development of their disease [32]; and that they have an allergic response to milk and meat [28].

The major strengths of this study are its population-based, prospective design and detailed information on dietary habits assessed both in the year 1987 and 1997. The value of a prospective study design cannot be emphasized enough, since any evaluations of dietary risk factors by a cross-sectional or retrospective study design infer a major risk of recall errors and recall bias, as well as a probability that the patients might change their dietary habits when struck by a chronic disabling disease [33]. The main limitation in this study is the identification of incident RA patients from national registers, which may introduce a statistical type-II error since some patients with RA may not be identified and censored. This therefore decreases the statistical power, rendering it more difficult to identify an association. It may also be noted that regular consumption of meat and dairy products was common in the studied population which limits the ability to assess the effect of a strict vegetarian or vegan diet on the risk of developing RA. Moreover, the fact that the study only consisted of women restricts the generalizability of our results.

## 5. Conclusions

Altogether, no clear association was observed between the dietary intake of meat and dairy products and the risk of developing RA in this large prospective cohort of women. It can be noted that further research may be needed to understand whether the consumption of meat and dairy products contributes to the development of RA among specific individuals or specific subgroups of patients.

## Figures and Tables

**Table 1:** Characteristics of 35,600 women born in 1914–1948 from the Swedish Mammography Cohort in 1997.

	**Total Consumption of Meat and Meat Products**			
	**≤4 Servings/Week**	**>4–7 Servings/Week**	**>7–10 Servings/Week**	**>10 Servings/Week**
N of women	6229	10,527	9583	9261
N of cases	62	97	112	97
Age (years)	63.83 (9.48)	61.36 (9.00)	60.46 (8.85)	61.23 (8.97)
Current smokers (%)	21.78	22.01	23.53	22.54
	**Total Consumption of Dairy and Dairy Products**			
	**≤3 Servings/Week**	**>3–4.5 Servings/Week**	**>4.5–6 Servings/Week**	**>6 Servings/Week**
N of women	7150	9775	7805	10,853
N of incident RA patients	78	103	76	111
Age (years)	60.32 (8.94)	61.52 (9.09)	61.78 (9.13)	62.11 (9.14)
Current smokers (%)	25.98	21.90	20.42	22.31

**Table 2:** Consumption of meat and meat products in 1997 and relative risk of developing rheumatoid arthritis among women in the Swedish Mammography Cohort during a 12 year follow-up period (January 2003–December 2014).

	No. of Newly Diagnosed Cases with RA	No. of Person Years	HR, Adjusted for Age	HR, Fully Adjusted Model *
**Meat, Overall**				
≤4 servings/week	62	64,230	Ref	Ref
>4–7 servings/week	97	113,706	0.84 (0.61–1.15)	0.88 (0.64–1.22)
>7–10 servings/week	112	104,390	1.04 (0.76–1.42)	1.13 (0.81–1.56)
>10 servings/week	97	99,131	0.96 (0.69–1.32)	1.08 (0.77–1.53)
**Red Meat**				
≤4 servings/week	83	87,288	Ref	Ref
>4–7 servings/week	120	125,334	0.96 (0.73–1.27)	1.01 (0.76–1.35)
>7–10 servings/week	89	89,256	1.00 (0.74–1.35)	1.08 (0.79–1.47)
>10 servings/week	76	79,579	0.97 (0.71–1.32)	1.08 (0.77–1.50)
**Processed Meat**				
≤1 serving/week	54	52,945	Ref	Ref
>1–3 servings/week	110	121,488	0.84 (0.60–1.16)	0.86 (0.62–1.20)
>3–6 servings/week	131	120,021	1.01 (0.73–1.39)	1.06 (0.76–1.46)
>6 servings/week	73	88,003	0.78 (0.55–1.11)	0.84 (0.59–1.22)
**Poultry**				
0 servings/week	47	52,122	Ref	Ref
≤1 serving/week	253	252,887	0.97 (0.71–1.33)	1.01 (0.73–1.40)
>1 serving/week	68	77,448	0.82 (0.56–1.19)	0.88 (0.60–1.31)

* Adjusted for age, alcohol intake, smoking, energy intake, dairy, and fish consumption.

**Table 3:** Consumption of dairy and dairy products in 1997 and relative risk of developing rheumatoid arthritis (RA) among women in the Swedish Mammography Cohort during a 12 year follow-up period (January 2003–December 2014).

	No. of Newly Diagnosed Cases with RA	No. of Person Years	HR, Adjusted for Age	HR, Fully Adjusted Model *
**Total Dairy**				
≤3 servings/week	78	77,304	Ref	Ref
>3–4.5 servings/week	103	105,043	0.99 (0.74–1.33)	1.03 (0.76–1.40)
>4.5–6 servings/week	76	83,777	0.92 (0.67–1.27)	0.99 (0.70–1.40)
>6 servings/week	111	115,333	0.98 (0.74–1.32)	1.09 (0.76–1.55)
**Milk**				
≤0.5 serving/week	121	125,297	Ref	Ref
>0.5–1 serving/week	67	62,227	1.14 (0.85–1.54)	1.15 (0.85–1.55)
>1–2 servings/week	95	104,788	0.99 (0.76–1.30)	1.00 (0.76–1.32)
>2 servings/week	85	89,145	1.07 (0.81–1.41)	1.07 (0.80–1.43)
**Cheese**				
≤1 servings/week	96	103,159	Ref	Ref
>1–2 servings/week	119	124,044	1.05 (0.80–1.37)	1.08 (0.82–1.42)
>2–4 servings/week	106	108,849	1.06 (0.81–1.40)	1.13 (0.84–1.51)
>4 servings/week	47	45,405	1.09 (0.77–1.55)	1.20 (0.81–1.76)

* Adjusted for age, alcohol intake, smoking, energy intake, meat and fish consumption.

**Table 4:** Long-term consumption of meat and dairy products between 1987 and 1997 and relative risk of developing rheumatoid arthritis (RA) among women in the Swedish Mammography Cohort during a 12 year follow-up period (January 2003–December 2014).

	Intake of Meat Products in 1997 *			
Intake of Meat Products in 1987 **	**<7 Servings/Week**		**≥7 Servings/Week**	
	**No. of Newly Diagnosed Cases with RA**	**HR (95% CI)**	**No. of Newly Diagnosed Cases with RA**	**HR (95% CI)**
<7 servings/week	155	Ref	69	1.02 (0.77–1.37)
≥7servings/week	48	0.80 (0.58–1.11)	96	0.98 (0.75–1.28)
	Intake of Dairy and Dairy Products in 1997 *			
Intake of Dairy Products in 1987 **	≤4.5 Servings/Week *		**>4.5 Servings/Week**	
	**No. of Newly Diagnosed Cases with RA**	**HR (95% CI)**	**No. of Newly Diagnosed Cases with RA**	**HR (95% CI)**
≤2.5 servings/week	108	Ref	64	1.19 (0.76–1.88)
>2.5 servings/week	72	1.05 (0.74–1.50)	123	1.34 (0.88–2.06)

Adjusted for age, alcohol intake, smoking, energy intake, meat/dairy (respectively), and fish consumption. Sub-analyses restricted to smokers and ex-smokers did not reveal any association (data not shown). * Divided by median consumption in the 1997 Food frequency questionnaire (96 questions). ** Divided by median consumption in the 1987 Food frequency questionnaire (67 questions).
